# Optimal Facility-Location

**DOI:** 10.6028/jres.111.008

**Published:** 2006-04-01

**Authors:** A. J. Goldman

**Affiliations:** Applied Math and Statistics Department, The Johns Hopkins University, Baltimore, MD 21218; National Institute of Standards and Technology, Gaithersburg, MD 20899

**Keywords:** mail sorting, mesometric facility, metric space, obnoxious facility, optimization

## Abstract

Dr. Christoph Witzgall, the honoree of this Symposium, can count among his many contributions to applied mathematics and mathematical operations research a body of widely-recognized work on the optimal location of facilities. The present paper offers to non-specialists a sketch of that field and its evolution, with emphasis on areas most closely related to Witzgall’s research at NBS/NIST.

## 1. Three Things, (LOC)^3^, Kroc

The present paper differs in some ways from most of the others presented at this Symposium: more reflective and expository than technical; more informal; and perhaps even a bit lighthearted, as befits an occasion celebrating the career and remarkable accomplishments of a distinguished colleague. Casting about for a means of introducing the particular class of problems to be discussed, and of testifying both to their importance and to the world’s recognition of that importance, I was reminded of a rhetorical question beloved by both property-conscious tycoons and academic geographers: “What are the three most important things in the success of a new supermarket (or gas station, or housing development)?” Quite likely you’ve heard the answer: “Location, location, location”.

So now the first two-thirds of this Section’s title has been justified. The site of my present main affiliation, The Johns Hopkins University, has active “locators” not only in my own Department, but also and very prominently in our sister Department of Geography and Environmental Engineering. I asked a friend, Professor Charles Revelle of that Department, about the origin of the rhetorical question and answer just given, and he was good enough to post it both to SOLA (the Section on Location Analysis of the Institute for Management Science) and to EWGLA (the EURO Working Group on Locational Analysis).

One respondent, presumably thinking of domiciles rather than commercial enterprises, attributed it “to the first cavemen”—and indeed having a cave located out of the paths of wind and rain, and beyond the leaping range of the saber-tooths (DID saber-tooths leap?), could well be essential for domestic bliss. A second one suggested the great hotelier Conrad Hilton. I would love to award the palm to Hilton, being biased by the fact that in his autobiography [4] he says that his two years of college math contributed mightily to his abilities for business analysis. But the preponderance of “votes” and supporting evidence points to Ray Kroc (that’s the “third third”), founder of McDonald’s; one cited source was [6], in which Kroc also points out that he’s basically in the real estate business, not the hamburger business. From there a further trail leads to one Luigi Salvaneschi, who rose from burger-flipping to a senior position at McDonald’s, a vice-presidency at Kentucky Fried Chicken, and the presidency of Blockbuster Entertainment; his allusion to Dante as a source of business acumen is reminiscent of the name of the restaurant (“The Flaming Inferno”) at which the Symposium’s dinner was held, but more immediately germane is the title of his popular business text [5].

## 2. Samizdats, 8388, Mail Must Go Through

Recall that the term “samizdat”, in the former Soviet Union, referred to a written work (often quite meritorious) which was not formally published. That description certainly applies to Witzgall’s major opus in facility location, the magisterial 1964 NBS Technical Report 8388 “Optimal Location of a Central Facility: Mathematical Models and Concepts” [10]. Neither suppressed by the authorities nor needing to be printed clandestinely, it still never saw “journal” or “treatise” publication, yet remains one of the most frequently-cited references in the field. (I was horrified to find that the NIST archives apparently retain only one copy of this classic, though at least accompanied by a 1992 note “DO NOT WEED”. I should hope not!) By the way, this was not the only one of Chris’s National Bureau of Standards (NBS) outputs to achieve “much-cited classic” status; the same is true of his 1973 Technical Note “A Performance Comparison of Labeling Algorithm for Calculating Shortest Paths”, co-authored with Symposium attendee Judith Gilsinn.

The origin of “8388” may be of interest. Back 40 years ago when it was written, the Post Office was an Executive Department, whose head was a Cabinet member; this “Postmaster-General” traditionally also served as director of patronage for whatever administration was in office, a role arising rather naturally from the sheer ubiquity of post offices and numbers of postal employees and jobs. But beyond politics, the nature of postal activities made them technological “naturals” for the application of engineering and analytical skills. In particular, the ever-growing volume of mail called loudly for aid by some kind of electro-mechanical sorting and processing system, and NBS engineers were actively involved in the design and evaluation of such systems. The routing and scheduling of mail transportation, both locally and between regions of the country, was also studied. There would need to be centralized facilities containing the large-scale sorting systems: mail collected locally would be brought to the nearby “sorting center”, separated out (i.e., sorted) by general destination, transported to the sorting center that served that destination, then (typically after a finer sorting) distributed locally to its “target” addresses.

Where should these massive sorting centers be located? Witzgall’s report, despite its more general title repeated above, was actually commissioned as the NBS mathematical response to this question, and the Post Office Department, via the broader NBS effort supporting it, was the “sponsor”. In order to convey what mathematics was already available to deal with such problems, what mathematical areas were involved, and what new research might be required, the issue was phrased in quite general terms. The document itself is a rather astonishing tour de force, containing a great variety of geometrical, analytical, computational, and even topological studies. This scope is symptomatic of the extent of Witzgall’s mathematical vision–and also the breadth of his cultural expertise, the report’s casual reference to A. Conan Doyle being of a piece with his knowledge about “Japanese temple mathematics”, the geometrical theorems appearing as “offerings” in some Japanese temples, an arcane but fascinating topic that was the subject of a Witzgall lecture at Johns Hopkins decades later.

## 3. Attractive, Obnoxious, Mesometric

The natural setting for optimal facility-location, apart from situations where command of a scenic view is of primary concern (are there other exceptions?), has long seemed to me to be a metric space, i.e. a “space” equipped with a function d(*x*, *y*) that satisfies certain natural conditions (like the “Triangle Inequality”) and is interpreted as giving the “distance” between points *x* and *y* in the space. Here “distance” is to be understood broadly, as a measure of disutility or impedance or “friction” to movement between the points. While Euclidean distances in the usual 2- or 3-dimensional space offer the most familiar and common example, other applications consider for instance the “shortest-path” distances between the nodes of a network.

[Some subtleties can arise in using such mathematical models. For example, in siting a recreation center in a tough urban neighborhood, the effective “distance” between a particular block and a prospective position for the “rec center” might well depend on whether or not a patron taking the “natural route” would need to pass through turf controlled by a gang hostile to the block’s home gang. Another example, with “disutility” now corresponding to travel time, is that the presence of strong head winds or water currents might lead to violations of the “natural condition” of symmetry (that d(*y*, *x*) = d(*x*, *y*)) and therefore require something more exotic than a garden-variety metric space.]

Although problems of optimal location are traced back to Toricello in the scholarship of “8388”, their popularity with geographers and spatial economists is generally taken to stem from Alfred Weber’s *Uber den Standort der Industrien* [9]. The “Weber Problem” is that of optimally locating an industrial plant in a planar region, relative to the positions of its suppliers and customers. If these entities (suppose there are *m* of them) are at the respective points *p_i_*, and would have respective interaction-intensities *w_i_* with the plant (e.g., in shipment-tons per year), then the plant’s location *x* is to be chosen to minimize the total interaction

$$f\left(x\right)=w_{1}\;d\left(x,p_{1}\right)+w_{2}\;d\left(x,p_{2}\right)+\ldots +w_{m}\;d\left(x,p_{m}\right).$$

Like the Weber Problem, the problems treated in “8388” generally have the property that the facility or facilities to be located are “attractive” ones; the entities that deal with them want them near, in order to reduce the cost or other disutility of interaction with them. (If one is dealing with facilities like hospitals or firehouses, which provide emergency services of some type, then the summation in the last display would typically be replaced by a “max” operator so that the aim is to ameliorate the situation of the least-well-served “client”.)

A near-exclusive emphasis on attractive facilities continued for a long time. But in the 1970s, concern about environmental considerations suggested study of optimal location for locations of the opposite type: those whose “obnoxious” nature (polluting, drawing undesirable visitors or even military attack) would make their appearance in your vicinity the subject of protest marches rather than the services of the Welcome Wagon. (It was for such facilities that the acronym NIMBY (“not in MY back yard”) was coined.) I had some responsibility for the kick-off of research in this area, via an early think-piece conference paper [3] and then the first doctoral dissertation I directed as a Hopkins faculty member [published in part as [Ting ]. These obnoxious facilities are ones people want as far away as possible, so that the minimizations arising for attractive facilities are replaced by maximizations.

Such a “three-letter change” (substitution of “max” for “min”) might seem innocuous, but in fact substantially increases the difficulty of actually carrying out the optimization: for example, now there can be local optima that are not global optima, so that “first-order” calculus-type techniques cannot suffice. And additional constraints appear; you might like to place the loathsome facility that handles your county’s garbage way “out at infinity”, or at least in the next county, but the next county is unlikely to permit that without a struggle or at least some quid pro quo. (New York City’s efforts over the years to find a home for its refuse is a fascinating saga of techno-political science; for an analog at the national level, “think nuclear wastes and Yucca Flats”.)

While mathematical and computational analysis for the siting of attractive facilities, and of obnoxious ones, remains a significant area of research and application, the field has begun to expand into a “third generation” of models. These deal with facilities that combine attractive and obnoxious attributes. Such “combination” can take various forms. For example, those likely to find employment at the new “skunk-works” might want it within convenient commuting range, while those whose sole interaction is to ingest its effluvia would have less neighborly inclinations. (See for example [1].) A second approach, adopted by another of my doctoral students [7] is to adopt the minimization criterion of the “attractive” case, but to overlay it with “forbidden region” constraints keeping the facility at arm’s length from those particularly sensitive to its offensive characteristics.

Still a third version of “combination” begins with a question like “how far from your home would you like the local firehouse to be?” Asking people to think aloud about this issue invariably elicits responses like “not too far, so that if I have a fire they can come quickly to my aid—but not too close, with so many fire-engines passing so frequently that my sleep would be siren-degraded, and my cat run over sooner or later”. The idea here is that the entities interacting with the facility each have some ideal distance at which they would like to lie, with penalties (disutilities) associated with both positive and negative deviations from this ideal. (Sounds like a geographer’s version of the “just right” theme in the “Goldilocks” story.) And one would like to situate the facility so as to minimize some weighted sum (or, perhaps, maximum), over the “inter-actors”, of these penalties. Consulting a colleague from the Hopkins Classics Department revealed the appropriate term for such facilities: “mesometric”, where as above “metric” means “distance”, while the prefix “meso” [as for example in “Mesopotamia” and “Mesozoic”) means “between”, often in the sense of “a happy medium”.

Optimal location problems for mesometric facilities turn out to be considerably harder than the “obnoxious” version, even for one-dimensional models. There’s a lot left to be done, but good initial progress including treatment of several “network” scenarios was made by my recent student Megan Deeney [2].

The following “non-geographical” interpretation of ideas like these, which adapts some of the literature for the “attractive” case, might provide some “feel” as to non-obvious possibilities for application. Suppose a firm is designing a new product to bring to market. The various attributes of that product (e.g., physical dimensions, expected lifetime, cost, etc.) can be regarded as coordinates in a “space”; different possible designs correspond to different possible points in that space, while already-existing products with which the new one will have to compete are represented by “given” points, perhaps weighted by the sizes of the market shares they command. One might want to situate the new product to be far enough (in this space) from the existing ones that it can really differentiate itself from them as an alternative to purchase, yet not so far as to seem a bizarre outlier not really competing to meet the (roughly) same set of needs and desires. In this context, the “optimal design” problem for the new product is “simply” the optimal location problem for the corresponding point in “design space”. (Some of the other literature along these lines speaks not of a firm designing a product, but rather of an unscrupulous politician selecting “stands” on various issues (the coordinates of the space) so as to form a “platform” optimally positioned relative to those already adopted by various opponents, and to those that would be most preferred by various interest groups of known strength.)
